# Ethanol extract of *Chrysanthemum zawadskii* Herbich induces autophagy and apoptosis in mouse colon cancer cells through the regulation of reactive oxygen species

**DOI:** 10.1186/s12906-019-2688-0

**Published:** 2019-10-21

**Authors:** Kwang-Youn Kim, Tae-Woo Oh, Hye-Jin Yang, Young-Woo Kim, Jin-Yeul Ma, Kwang-Il Park

**Affiliations:** 1 Korean Medicine (KM) Application Center, Korea Institute of Oriental Medicine (KIOM), Daegu, 41062 Republic of Korea; 2 Department of Herbal Formula, Medical Research Center (MRC-GHF), College of Oriental Medicine, Daegu Haany University, Gyeongsan, 38610 Republic of Korea

**Keywords:** *Chrysanthemum zawadskii* Herbich, Colon cancer, Apoptosis, Autophagy, Reactive oxygen species

## Abstract

**Background:**

Recent research has suggested that autophagy can provide a better mechanism for inducing cell death than current therapeutic strategies. This study investigated the effects of using an ethanol extract of Chrysanthemum zawadskii Herbich (ECZ) to induce apoptosis and autophagy associated with reliable signal pathways in mouse colon cancer CT-26 cells.

**Methods:**

Using ECZ on mouse colon cancer CT-26 cells, cell viability, annexin V/propidium iodide staining, acridine orange staining, reactive oxygen species (ROS) and western blotting were assayed.

**Results:**

ECZ exhibited cytotoxicity in CT-26 cells in a dose-dependent manner. ECZ induced apoptosis was confirmed by caspase-3 activation, poly (ADP-ribose) polymerase cleavage, and increased production of reactive oxygen species (ROS). Furthermore, it was shown that ECZ induced autophagy via the increased conversion of microtubule-associated protein 1 light chain 3II, the degradation of p62, and the formation of acidic vesicular organelles. The inhibition of ROS production by N-Acetyl-L-cysteine resulted in reduced ECZ-induced apoptosis and autophagy. Furthermore, the inhibition of autophagy by 3-methyladenine resulted in enhanced ECZ-induced apoptosis via increased ROS generation.

**Conclusion:**

These findings confirmed that ECZ induced ROS-mediated autophagy and apoptosis in colon cancer cells. Therefore, ECZ may serve as a novel potential chemotherapeutic candidate for colon cancer.

## Background

Colon cancer is the third most common cancer in men and the second most common cancer in women worldwide [1]. Early diagnosis with colonoscopy and the removal of precancerous lesions has resulted in a recent decline in the incidence of colon cancer in the United States; however, its incidence is increasing in Asia and Eastern Europe [2]. In Korea, despite the development of various treatment methods, colon cancer has now become the fourth leading cause of overall mortality, and its incidence is still increasing in both men and women.

Autophagy affects a wide range of processes, including homeostasis, developmental process, immune function, aging, and various cellular function [3]. Autophagy is a catabolic process which involves the degradation of the large protein complexes and dysfunctional organelles. These components are sequestered and transported to lysosomes for degradation as a cytoprotective mechanism [4]. Also, when cellular stress is extensive, autophagy acts degradation and recycling of process by the accumulation of acidic vesicular organelles (AVOs) through an alternative cell-death pathway as cytotoxic function [5, 6]. Therefore, the dysfunction of autophagy can affect the incidence and treatment of diseases such as cancer [7]. Recent reports have proposed autophagy as a novel strategy for cancer therapy [8, 9]. However, the action of autophagy in cancer is highly complex and affected by genetic differences [10, 11]. When apoptosis is excessive or deficient in the spontaneous destruction pattern of cells, this can contribute to the growth and recurrence of ischemia, neurodegenerative disease, autoimmune disease, viral infection, and tumors [2, 12]. Recently, the complex interactions between autophagy and apoptosis have received attention, with studies showing that apoptosis can sometimes act as an inhibitor or inducer of autophagy, thus resulting in changed resistance to many anticancer drugs or to a clinical application [13, 14]. Further studies are needed on the interaction between autophagy and apoptosis under various conditions [15].

*Chrysanthemum zawadskii* Herbich (CZ) is a perennial herb from the family Asteraceae, which is grown in countries including China, Russia, Mongolia, and Japan [16]. Extracts of CZ have been used in traditional medicine and as a tea in Korea and China. CZ has been shown to have effective therapeutic and medicinal properties, including antimicrobial, antioxidant, and antimycotic activity [17–19]. Linarin, one of its physiologically active agents, has been reported to exhibit antiinflammatory, antipyretic, hepatoprotective, antibacterial, anticancer, and antioxidant activity [20–22]. However, although the beneficial and pharmacological effects of CZ are established, the molecular mechanisms underlying its anticancer effects in colon cancer remain unknown. Therefore, the aim of the present study was to investigate the chemotherapeutic effects of an ethanol extract of CZ (ECZ) and to elucidate the interrelated mechanisms involving apoptosis and autophagy in mouse colon cancer CT-26 cells. The results showed that the production of reactive oxygen species (ROS) by ECZ may offer a therapeutic strategy to improve the treatment of colon cancer through the relationship between autophagy and apoptosis.

## Materials and methods

### Reagents

Chlorogenic acid and 3,5-di-caffeoylquinic acid were purchased from Sigma-Aldrich (St. Louis, MO, USA). Luteolin was obtained from Faces Biochemical Co., Ltd. (Wuhan, China). HPLC-grade acetonitrile was purchased from Thermo Fisher Scientific (Pittsburgh, PA, USA), and LC/MS-grade formic acid was purchased from Sigma-Aldrich. The ultrapure water used in the HPLC analysis was prepared using a Puris-Evo UP Water system with Evo-UP Dio VFT and Evo-ROP Dico20 (Mirae ST Co., Ltd., Anyang, Gyeonggi-do, Korea). Dulbecco’s modified Eagle’s medium (DMEM), penicillin/streptomycin and fetal bovine serum (FBS) were obtained from Hyclone (Logan, UT, USA). Acridine orange (AO), dichlorodihydrofluorescein diacetate (DCF-DA), 3-methyladenine (3-MA), and N-Acetyl-L-cysteine (NAC) were purchased from Sigma-Aldrich. Cell counting kit (CCK)-8 assays and FITC Annexin V-Apoptosis Detection Kit were obtained from Dojindo Molecular Technologies, Inc. (Rockville, MD, USA) and BD Biosciences (San Jose, CA, USA), respectively. Primary antibodies against Bax, Bcl-2, caspase 3, poly (ADP-ribose) polymerase (PARP), microtubule-associated protein 1 light chain-3B (LC3B), p62/SQSTM1, and β-actin were purchased from Cell Signaling Technology (Danvers, MA, USA), and Santa Cruz Biotechnology (Santa Cruz, CA, USA), respectively. The antimouse IgG and goat antirabbit secondary antibodies were purchased from Enzo Life Science (Farmingdale, NY, USA).

### Preparation of standard solutions and ECZ

Standard stock solutions in methanol (1 mg/ml) of chlorogenic acid, 3,5-di-caffeoylquinic acid, and luteolin were prepared. The standards were mixed from the stock solutions, and then freshly prepared by serial dilution in methanol to plot the calibration curves. The final concentrations of the calibration samples were in the range 25–400 μg/ml for chlorogenic acid and 3,5-di-caffeoylquinic acid, and 1.25–20.00 μg/ml for luteolin. CZ was purchased from Yeongcheon Oriental Herbal Market (Yeongcheon, Korea) and was authenticated by Professor Ki Hwan Bae, a medical botanist at the College of Pharmacy, Chungnam National University, Republic of Korea. A voucher (No. 203) was deposited at the Korean Medicine Application Center Korea Institute of Oriental Medicine in Daegu, Republic of Korea. Dried CZ (30 g) was ground to a fine powder, added to 300 ml of 70% ethanol, and then extracted by shaking it in an incubator at 100 rpm for 24 h at 40 °C. The extract was then filtered through a 150 μm testing sieve (Retsch, Haan, Germany), evaporated, concentrated through lyophilization, and then stored at − 20 °C (yield 13.83%). For the experiments, ECZ powder (10 mg) was dissolved in 1 ml of deionized distilled water (v/v) and filtered through a 0.22 μm disk filter.

### High performance liquid chromatography (HPLC) analysis

The three components in ECZ were identified and quantified by HPLC via a previously reported method [23, 24]. In this study, the HPLC analysis was performed using a Dionex UltiMate 3000 system (Dionex Corp., Sunnyvale, CA, USA) equipped with a binary pump, an auto-sampler, a column oven and a diode array UV/Vis detector (DAD). System control and data analysis were performed using Dionex Chromelon software. The three components were eluted in a gradient system based on 0.1% formic acid in deionized water (solvent A) and acetonitrile (solvent B). To improve the chromatographic separation capacity, the gradient elution system was programmed as follows: 5–12% B, 0–5 min; 12–15% B, 5–7 min; 15% B, 7–12 min; 15–20% B, 12–14 min; 20% B, 14–20 min; 20–60% B, 20–35 min; 60–90% B, 35–40 min; 90% B, 40–45 min; 90–95% B, 45–46 min; 5% B, 46–60 min at a flow rate of 1.0 ml/min. The components were separated on a Xbridge C18 column (250 × 4.60 mm, 5 μm; Waters, Milford, MA, USA), and the column oven temperature was kept at 40 °C. The injection volume was 5 μl. The detection wavelengths for the three components were set at 220, 254, 320 and 365 nm.

### Cell culture

The mouse colon cancer CT-26 and human colon cancer HT-29 cell lines were obtained from American Type Culture Collection (Manassas, VA, USA). The cells were cultured using DMEM containing 10% FBS, 100 units/ml penicillin and 100 μg/ml streptomycin at 37 °C in a humidified atmosphere with 5% CO_2_.

### Cell viability

The cytotoxicity of ELT on CT-26 cells was calculated using CCK-8 assay. The cells were seeded at 1 × 10^4^ cells/well in a 96-well plate. After incubation for 24 h, the cells in each well were treated with ELT at specific concentrations for 24 h. A CCK-8 assay was then performed in accordance with the manufacturer’s instructions. Absorbance was determined at 450 nm on a VERSAmax microplate reader (Molecular Devices, Sunnyvale, CA, USA). Cell viability was calculated relative to untreated controls as follows: viability (% control) = 100 × absorbance of treated sample/absorbance of control.

### Annexin V/propidium iodide (PI) assay

To verify the induction of apoptosis, CT-26 and HT-29 cells were cultured at a density of 1 × 10^4^ cells and treated with ECZ for 24 h. The cells were then collected and double stained with Annexin-V-FITC and PI (BD Biosciences), following the manufacturer’s instructions. Apoptotic cells were determined by flow cytometry (Becton Dickinson Co.) and the percentages of apoptotic cells were calculated using Cell Quest software (Becton Dickinson Co.).

### Detection of acidic vesicular organelles

To quantify the number of AVOs, CT-26 and HT-29 cells were cultured at a density of 1 × 10^4^ cells/well in 6-well plates. After treatment with ECZ for 24 h, the cells were stained with AO (1 μg/ml) at 37 °C for 20 min in the dark. The cells were then washed with PBS, analyzed by a flow cytometry (Becton Dickinson Co.), and quantified using Cell Quest software (Becton Dickinson Co.).

### Measurement of intracellular ROS generation

To determine intracellular ROS production, CT-26 cells were cultured in 6-well plates at a density of 1 × 10^4^ cells/well. The cells were treated with ECZ and incubated with DCFH-DA (10 μM) at 37 °C for 30 min and then washed twice with PBS. For each experiment, the cells were analyzed by flow cytometry (Becton Dickinson Co.), and quantified using Cell Quest software (Becton Dickinson Co).

### Western blotting

Cell extracts were prepared by incubating in lysis buffer [150 mM NaCl, 10 mM Tris (pH 7.4), 5 mM EDTA (pH 8.0), 1% Triton X-100, 1 mM PMSF, 20 μg/ml aprotinin, 50 μg/ml leupeptin, 1 mM benzidine, and 1 mg/ml pepstatin]. For separation using sodium dodecyl sulfate–polyacrylamide gel electrophoresis, 50 μg of proteins were loaded onto 12–15% gel and transferred to a polyvinylidene fluoride membrane. After blocking with TBS-T buffer [20 mM Tris (pH 7.4), 150 mM NaCl, and 0.1% Tween 20] containing 5% skim milk, the membranes were incubated with primary and secondary antibodies, separately. The membranes were then washed with TBS-T buffer and visualized with ECL Western blotting detection reagents. The density of each band was determined with a fluorescence scanner (LAS 3000, Fuji Film) and analyzed with Multi Gauge V3.0 software.

### Statistical analysis

Experiments were repeated to obtain three sets of consistent results. Unless otherwise stated, data are expressed as the mean ± standard deviation of the mean. ANOVA was used to compare experimental and control values. Comparisons between multiple groups were performed using Tukey’s multiple comparison tests, with the results considered statistically significant at ^***^ *p < 0.001*.

## Results

### HPLC analysis of ECZ

**Fig. 1:**
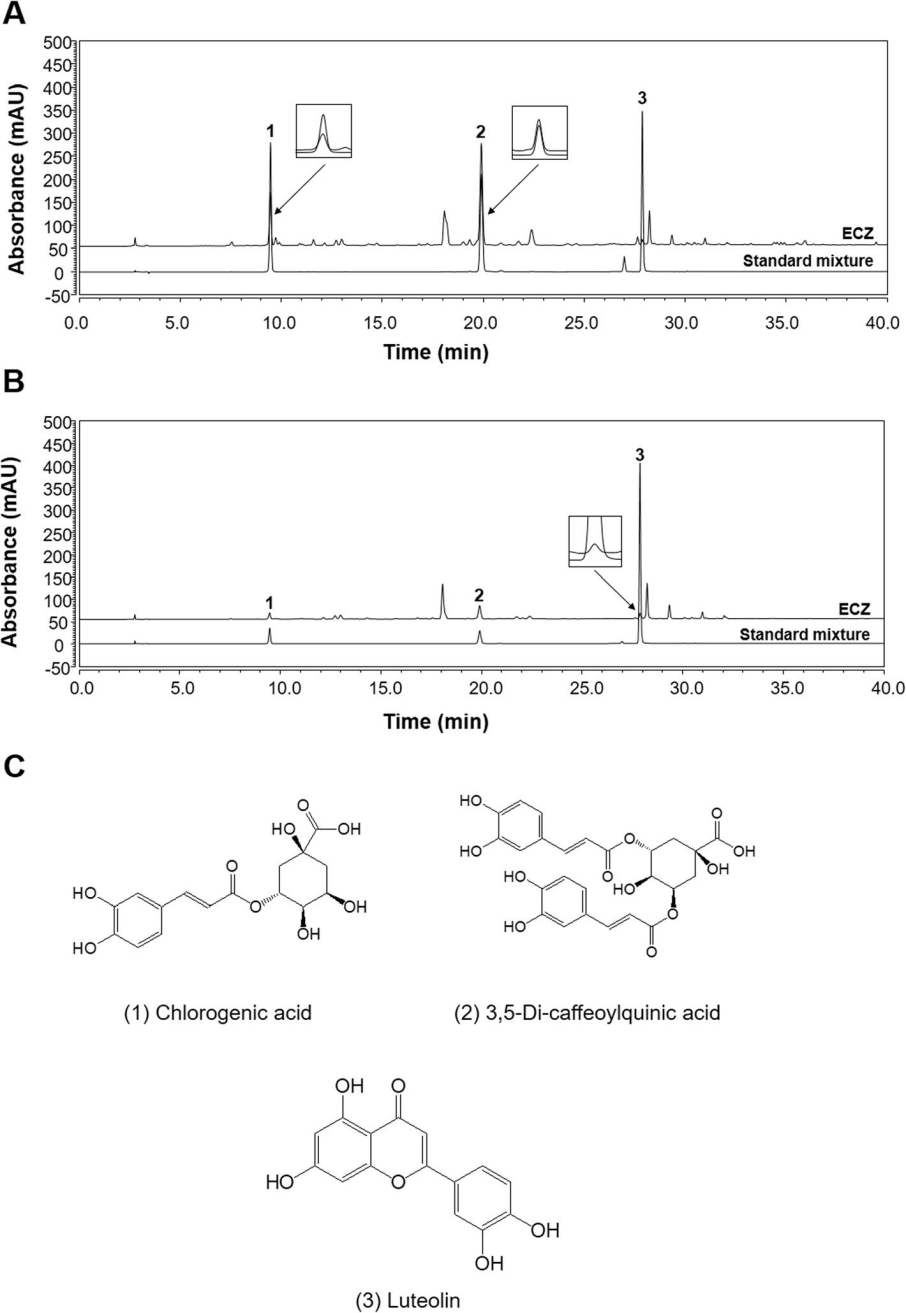
High-performance liquid chromatography (HPLC) chromatograms of ethanol extracts of Chrysanthemum zawadskii Herbich (ECZ) and the chemical structures of three markers. For ECZ, the markers were identified at wavelengths of (**a**) 320 and (**b**) 365 nm by using HPLC and a diode array UV/Vis detector. **c** The chemical structures of three markers of ECZ. (1) chlorogenic acid, (2) 3,5-di-caffeoylquinic acid, and (3) luteolin

The HPLC chromatograms for ECZ and a mix of the three standard components are shown in Fig. 1. The peaks for the three components in ECZ were identified by comparing their retention times, ultraviolet (UV) spectra, and chromatogram patterns with those of the standard mix. The target components were well separated and showed good selectivity, with no interference from other components within 40 min. The retention times of the compounds in the chromatogram of the standard mix were 9.45 (chlorogenic acid), 19.89 (3,5-di-caffeoylquinic acid), and 27.86 min (luteolin). Under the same conditions, the retention times of the observed three components in ECZ were 9.46 min (1), 19.89 min (2), and 27.87 min (3), respectively. The UV wavelength for the HPLC was optimized according to the UV spectrum and maximum absorption for each standard component; the wavelength was set at 320 nm for detecting the first two components and 365 nm for detecting the third. Five different concentrations of the standard solutions for calibration were analyzed in triplicate. The calibration curve for the quantitative analysis was obtained by plotting the concentration of each component versus the peak area; this showed good linearity (r^2^ ≥ 0.9997) (Table 1). The amounts of the three components in ECZ were analyzed by applying regression equations calculated from the calibration curves. The results of the quantitative analysis are summarized in Table 2.

**Table 1:** Calibration curves of three analytes using HPLC-DAD

No.	Components	Linear range (μg/mL)	Regression Equation ^a)^	Correlation coefficient (R^2^) ^b)^
1	Chlorogenic acid	25.00–400.00	y = 0.2428x + 1.9062	0.9997
2	3,5-Di-caffeoylquinic acid	25.00–400.00	y = 0.2843x - 1.6878	0.9999
3	Luteolin	1.25–20.00	y = 0.3257x + 0.0543	0.9999

a) y: peak area, x: concentration (μg/mL)

b) Regression coefficient (*n* = 3)

**Table 2:** The amounts of three analytes in ECZ using HPLC-DAD

No.	Components	*t*_*R*_	μg/mL	μg/mg	SD	RSD, %
1	Chlorogenic acid	9.46	38.13	3.81	0.22	1.96
2	3,5-Di-caffeoylquinic acid	19.89	99.43	9.94	0.78	2.43
3	Luteolin	27.87	3.38	0.34	0.02	1.96

### ECZ inhibited proliferation and triggered apoptosis in mouse colon cancer CT-26 cells

**Fig. 2:**
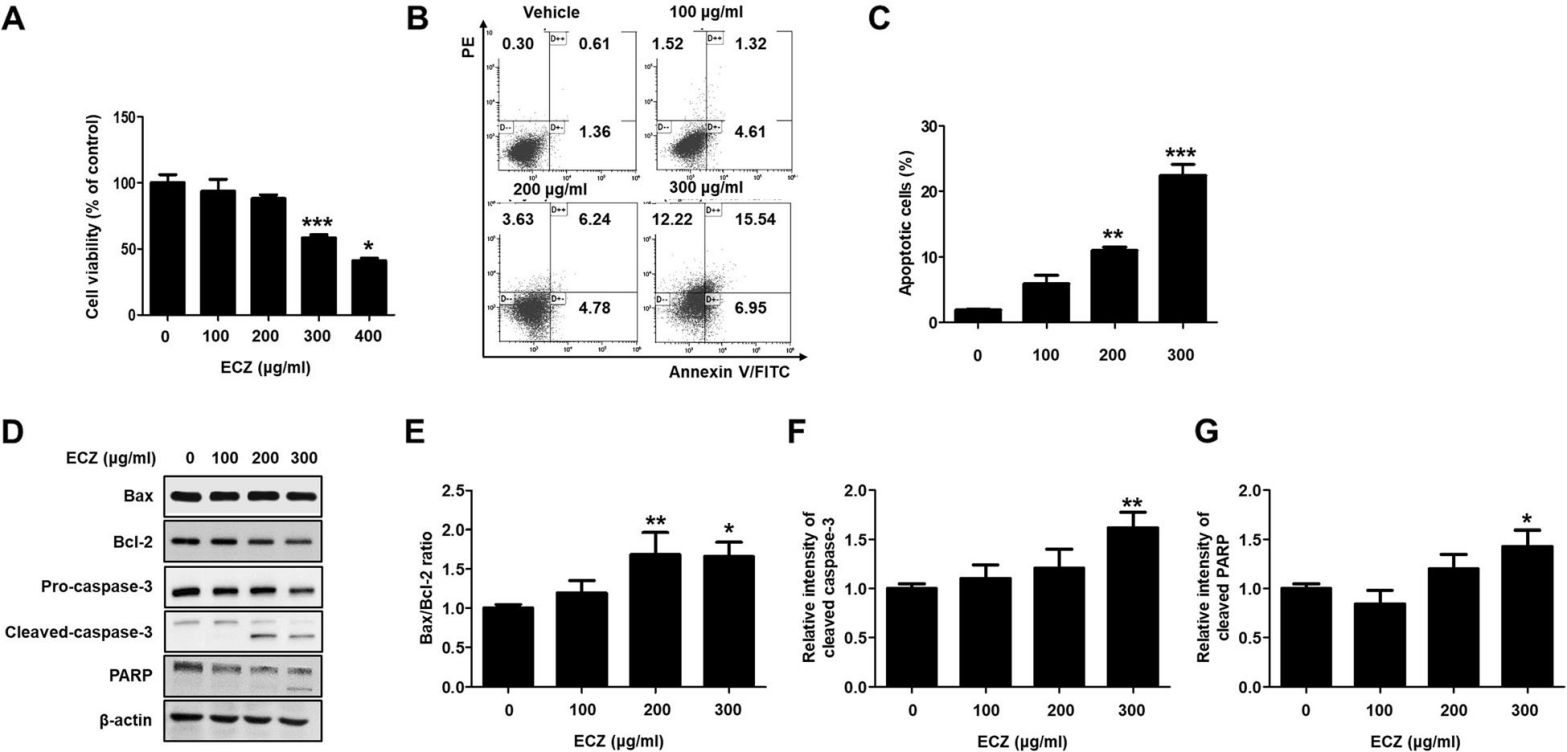
Ethanol extracts of Chrysanthemum zawadskii Herbich (ECZ) triggered apoptosis in mouse colon cancer CT-26 cells. **a** Cell viability. **b** Apoptosis induction. **c** Representive graph of apoptotic cells. **d** Expression of representative apoptosis-related proteins. **e** Bax/Bcl-2 ratio. **f** Relative fold change of cleaved caspase-3. **g** Relative fold changes of cleaved PARP. The cells were treated with various concentrations (100–300 μg/ml) of ECZ for 24 h. Cell viability was determined using a standard CCK-8 assay. Cell viability is represented as the percent of relative absorbance relative to the controls. Apoptosis induction was evaluated using annexin V/propidium iodide (PI) staining and flow cytometry. Total cell lysates were analyzed by Western blot analysis. Data are presented as the mean ± standard deviation for at least three independent experiments. *p < 0.05, **p < 0.01, ***p < 0.001

We examined the impact on cell viability of mouse colon cancer CT-26 cells to ECZ. Exposure significantly reduced the viability of CT-26 cells in a dose-dependent manner (Fig. 2a). To examine the anticancer effects of ECZ in CT-26 cells, we quantitatively verified apoptosis by using Annexin V-FITC/propidium iodide (PI) staining. After treatment with 200 and 300 μg/ml of ECZ for 24 h, flow cytometry analysis showed significantly increased in early and late apoptotic CT-26 cells from 2.02% to 10.30 and 15.05%, respectively (Fig. 2b and c). Addition, we confirmed that ECZ treatments also induced apoptosis in HT-29 cells (Additional file 1: Fig. S1A). To investigate the mechanism for ECZ-induced apoptosis in CT-26 cells, the expression levels of apoptosis-related proteins were evaluated by western blot analysis. ECZ exposure did not affect the expression of Bax, but reduced the expression of Bcl-2, and Bcl-2/Bax ratio was significantly reduced (Fig. 2d and e). Furthermore, ECZ reduced the pro-form of caspase-3 and increasingly induced the cleavage of capspase-3 and PARP in a dose-dependent manner (Fig. 2d, f and g). These results indicated that ECZ-induced apoptosis was functionally associated with caspase-3 dependent pathways.

### ECZ induced autophagy in CT-26 cells

**Fig. 3:**
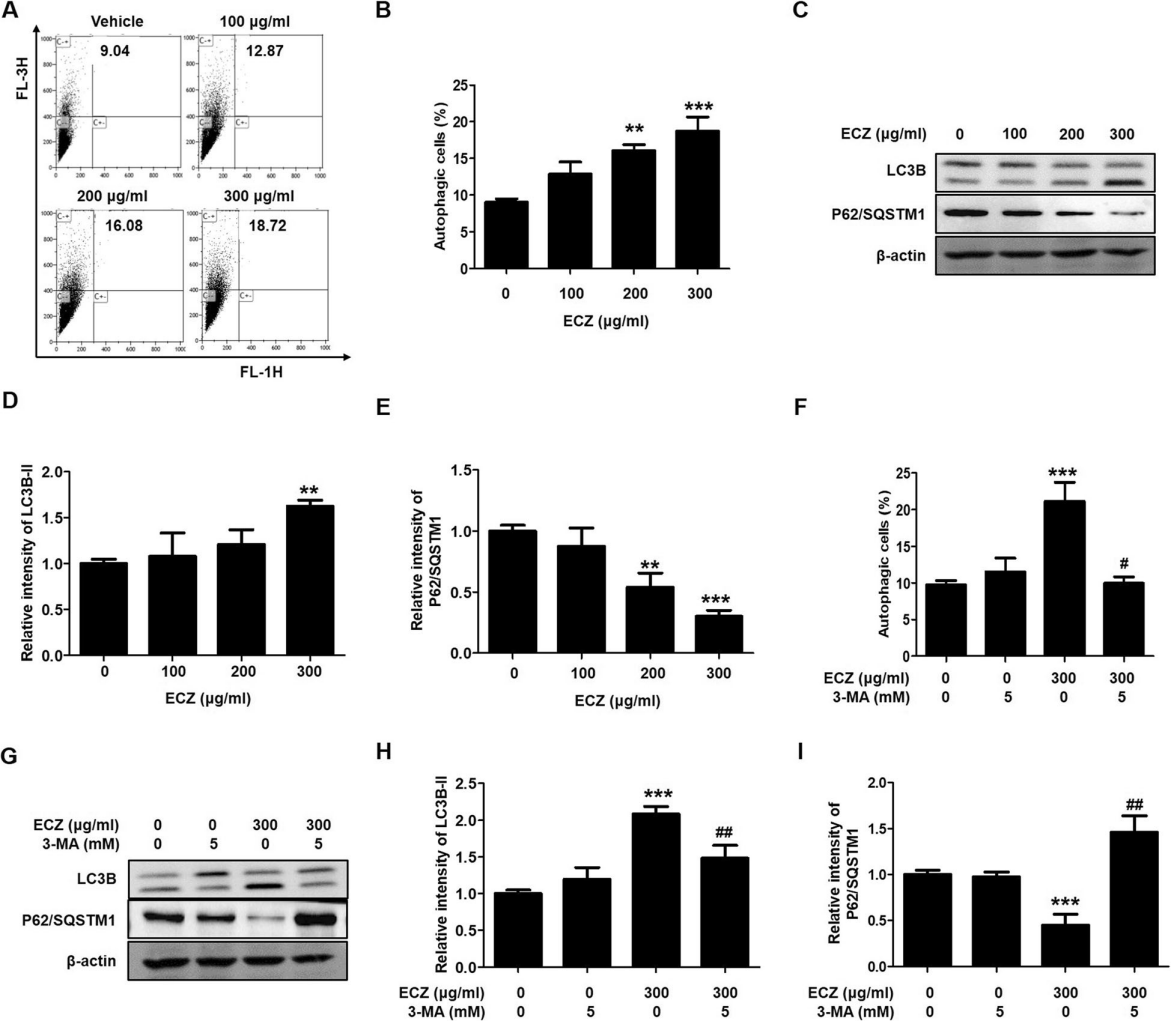
Ethanol extracts of Chrysanthemum zawadskii Herbich (ECZ) induced autophagy in mouse colon cancer CT-26 cells. **a** Detection of acidic vesicular organelles (AVOs). **b** Representive graph of autophagic celss. Cells were treated with various concentrations (100–300 μg/ml) of ECZ for 24 h and stained with 1 μM acridine orange (AO) at 37 °C in the dark for 20 min and then analyzed by flow cytometry. **c** Expression of LC3B and p62/SQSTM1. **d** Relative fold change of LC3B-II. **e** Relative fold change of p62/SQSTM1. Total cell lysates were analyzed by Western blotting. **f** Pretreatment with 3-MA reversed the ECZ-induced increase in autophagy. **g** Effect of 3-MA on LC3B and p62/SQSTM1 expression. **h** Relative fold change of LC3B-II. (I) Relative fold change of p62/SQSTM1. Cells were pretreated with 1 mM 3-MA prior to 1 h and AO-stained cells were evaluated by flow cytometry. Total lysates were subjected to SDS-PAGE for Western blot analysis. Data are presented as the mean ± standard deviation for at least three independent experiments. **p < 0.01, ***p < 0.001 vs. the control group; #*p* < 0.05, ^##^p < 0.01 vs. the ECZ treated group

We confirmed the effects of ECZ regarding the induction of autophagy by using AO staining. After treatment with 200 and 300 μg/ml of ECZ for 24 h, there were significantly increases in the accumulation of acidic vesicular organelles (AVOs) from 9.04 to 16.08% and 18.72%, respectively (Fig. 3a and b). Addition, we confirmed that ECZ treatments also induced autophagy in HT-29 cells (Additional file 1: Figure S1B). Furthermore, there were clear dose-dependent increased in the expression levels of LC3 conversion forms and p62/SQSTM1 (which are autophagy-specific markers) in ECZ-treated CT-26 cells (Fig. 3c, d and e). To reconfirm the ECZ-induced autophagy, we evaluated the formations of AVOs and the expression levels of LC3 conversion forms and p62/SQSTM1 by using the autophagy inhibitor 3-MA. The levels of formation of AVOs in the presence or absence of 3-MA; were 9.97 or 21.13%, respectively (Fig. 3f). Furthermore, the expression of LC3 conversion forms and p62/SQSTM1 significantly reverted when ECZ was combined with 3-MA compared with ECZ alone (Fig. 3g, h and i). These results indicated that ECZ induced autophagy by increasing autophagosome formations in CT-26 cells.

### ECZ-induced apoptosis and autophagy were regulated by ROS production in CT-26 cells

**Fig. 4:**
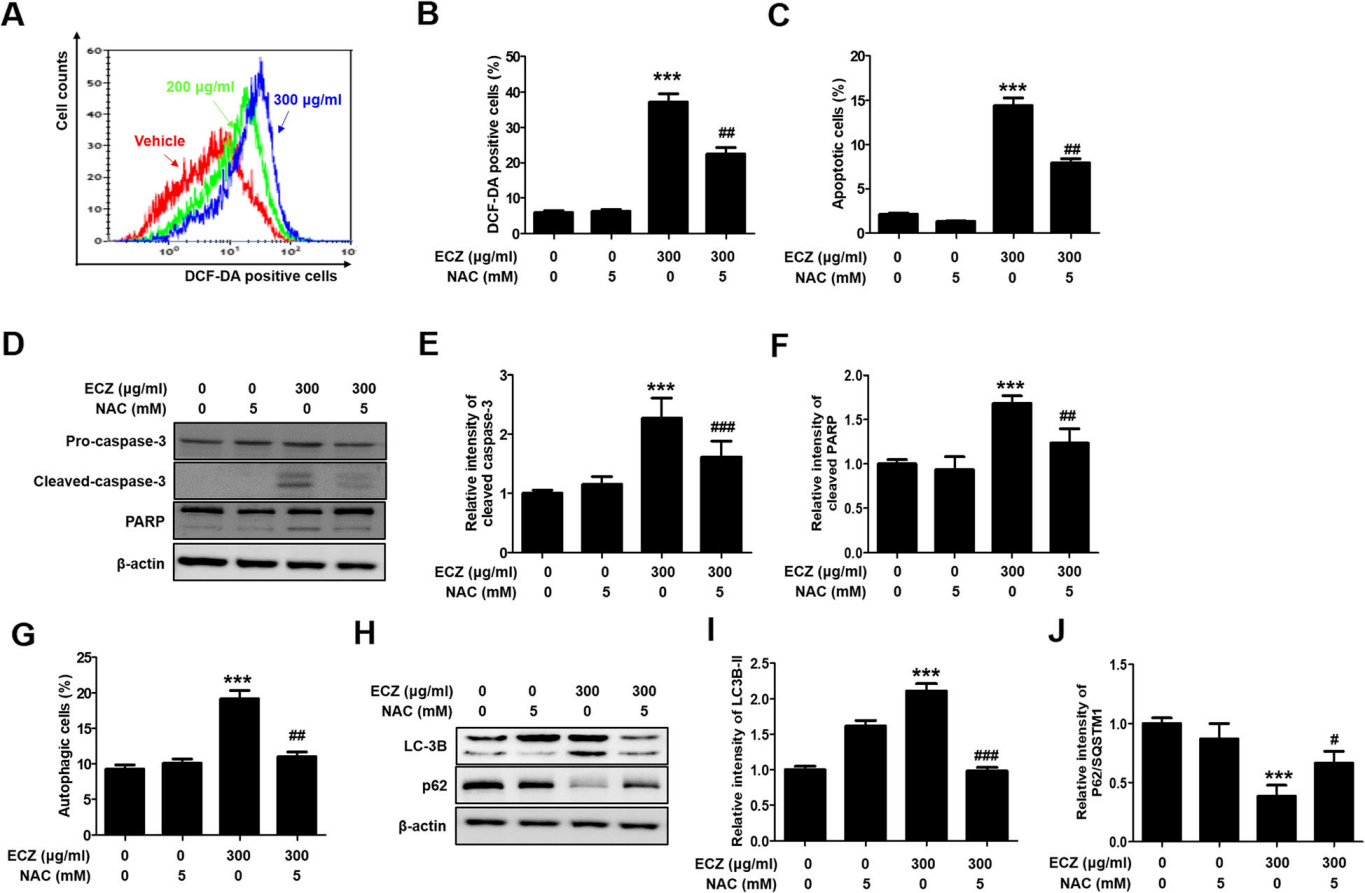
Reactive oxygen species (ROS) regulated apoptosis and autophagy induced by ethanol extracts of Chrysanthemum zawadskii Herbich (ECZ) in mouse colon cancer CT-26 cells. **a** Representative histogram of intracellular ROS levels. Cells were treated with various concentrations (200–300 μg/ml) of ECZ for 24 h, and dichlorodihydrofluorescein diacetate (DCF-DA) fluorescence intensity was detected by flow cytometry. **b** N-Acetyl-L-cysteine (NAC) attenuated the increased ROS production induced by ECZ. (C) NAC attenuated apoptosis induced by ECZ. **d** Effects of NAC on procaspase-3 and poly (ADP-ribose) polymerase (PARP) expression. **e** Relative fold change of cleaved caspase-3. **f** Relative fold change of cleaved PARP. **g** Effects of NAC on autophagy. **h** Effects of NAC on LC3B and p62/SQSTM1 expression. **i** Relative fold change of LC3B-II. **j** Relative fold change of p62/SQSTM1. Cells were treated with 300 μg/ml of ECZ for 24 h prior to 1 h incubation with 1 mM NAC. ROS production, apoptotic cells, and autophagic cells were detected by flow cytometry. Total lysates were subjected to SDS-PAGE for Western blot analysis. Data are presented as the mean ± standard deviation for at least three independent experiments. ***p < 0.001 vs. the control group; ^#^*p* < 0.05, ^##^*p* < 0.01, ^###^p < 0.001 vs. the ECZ treated group

Several recent studies have demonstrated that ROS plays an important role in the induction of apoptosis and autophagy [25, 26]. Thus, we used flow cytometry with DCF-DA staining to examine whether there was a relationship between ROS production and ECZ-induced apoptosis and autophagy in CT-26 cells. ROS production was significantly increased in a dose-dependent manner (Fig. 4a); this was reversed by pretreatment with NAC, which is a ROS scavenger (Fig. 4b). We then examined the effects of NAC on the crosstalk of ROS production between ECZ-induced apoptosis and autophagy. Pretreatment with NAC reduced both apoptotic cells and autophagic cells in CT-26 cells (Fig. 4c and g). Addition, we confirmed that pretreatment with NAC reduced both apoptotic cells and autophagic cells in HT-29 cells (Additional file 1: Figure S1C and 1D). Consistent with the flow cytometry results, NAC reversed the increased expression of procaspase-3, PARP, LC3 conversion, and p62/SQSTM1 (Fig. 4d, e, f, h, i and j). These results indicated that ROS played a critical role not only in ECZ-triggered apoptosis via caspase-dependent pathways but also in autophagy via LC3-II expression and AVOs accumulation.

### Inhibition of autophagy-enhanced ECZ-induced apoptosis via increased ROS production in CT-26 cells

**Fig. 5:**
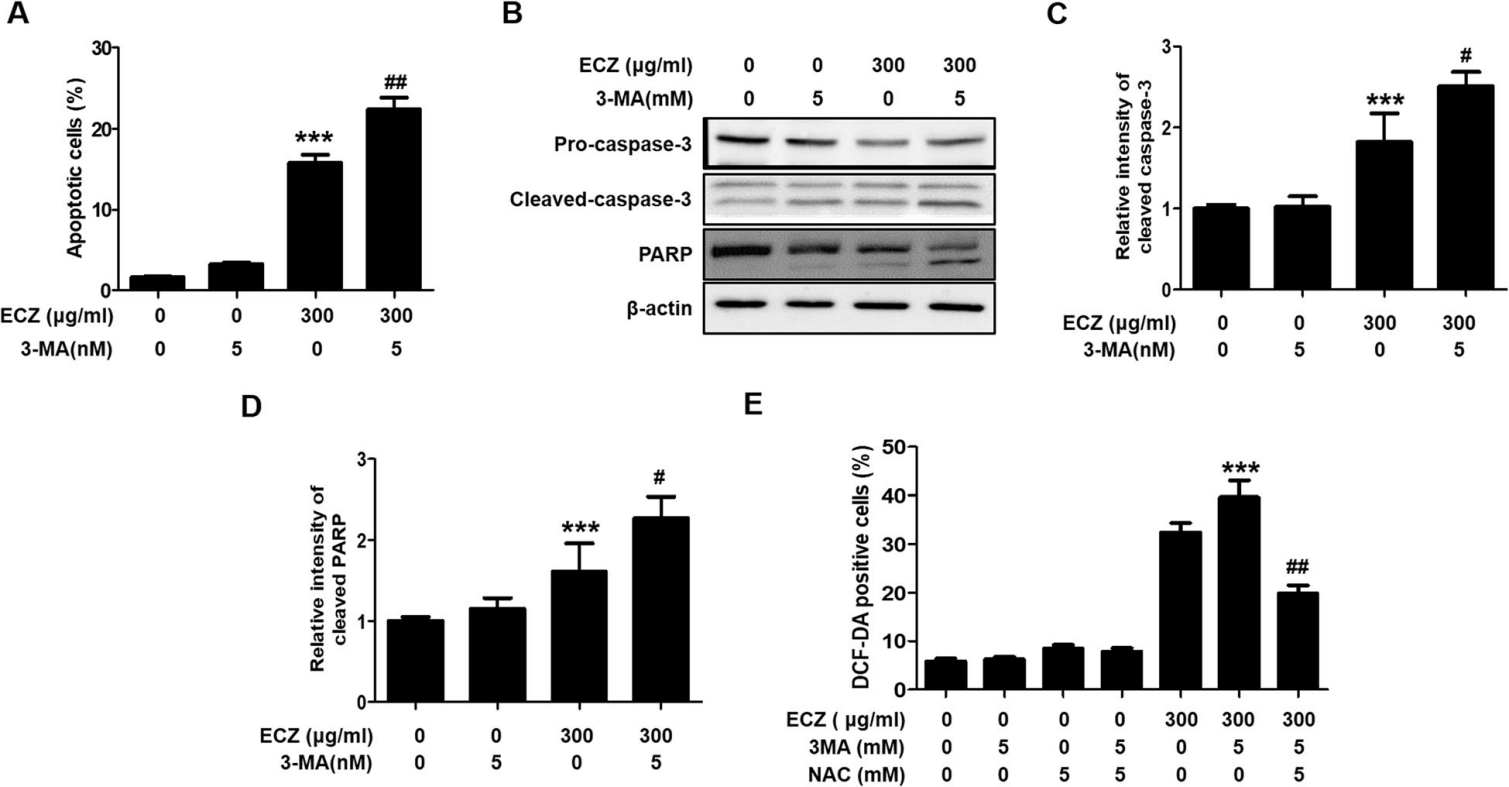
Inhibition of autophagy enhanced apoptosis induced by ethanol extracts of Chrysanthemum zawadskii Herbich (ECZ) by increasing the production of reactive oxygen species (ROS) in mouse colon cancer CT-26 cells. **a** Treatment with 3-methyladenine (3-MA) enhanced ECZ-induced apoptosis. **b** Effects of 3-MA treatment on caspase-3 and poly (ADP-ribose) polymerase (PARP) expression. **c** Relative fold change of cleaved caspase-3. **d** Relative fold change of PARP. Cells were pretreated with 5 mM 3-MA prior to 1 h. Apoptotic cells were evaluated by flow cytometry, and the proteins were detected by Western blot analysis. **e** Effects of 3-MA on ROS production. Cells were treated with 300 μg/ml ECZ for 24 h prior to 1 h incubation with 5 mM 3-MA or/and 5 mM NAC. ROS production was evaluated by flow cytometry. Data are presented as the mean ± standard deviation for at least three independent experiments. ***p < 0.001 vs. the control group, #p < 0.05, ^##^p < 0.01 vs. the ECZ treated group

To establish whether the inhibition of autophagy affected ECZ-induced apoptosis in CT-26 cells, we used flow cytometry with annexin-V-FITC staining after pretreatment with 3-MA to evaluate the induction of apoptosis. There was a greater increased in apoptotic cells among cells treated with a combination of ECZ and 3-MA than in cells treated with ECZ alone in CT-26 cells (Fig. 5a). Addition, we confirmed that pretreatment with 3-MA decreased ECZ-induced autophagy, but increased ECZ-induced apoptotic cells in HT-29 cells (Additional file 2: Figure S2A and S2B). Furthermore, there were greater increases in cleavage forms of caspase-3 and PARP proteins with the combination ECZ and 3-MA than with ECZ alone, thus indicating that the induction of apoptosis was further enhanced when autophagy was blocked with 3-MA (Fig. 5a-d). Thereafter, to evaluate whether the inhibition of autophagy affected the production of ROS, we analyzed ROS levels in CT-26 cells after treatment with ECZ and 3-MA. ROS production was significantly increased in cells treated with the combination of ECZ and 3-MA compared with those treated with ECZ alone (Fig. 5e), and this increase in ROS production was clearly attenuated by treatment with NAC (Fig. 5e). These results indicated that autophagy played a protective role against the induction of apoptosis in CT-26 cells by ECZ and that increasing ROS production by inhibiting autophagy enhanced the induction of apoptosis, consistent with the results in Fig. 5a.

## Discussion

CZ has been reported to have diverse effects on several diseases, including pneumonia, bronchitis, coughs, colds, pharyngitis, bladder disease, hypertension, liver disease, and gastrointestinal disorders [27–29]. However, the molecular mechanisms underlying ECZ’s anticancer effects are not yet understood. The aim of this study was to investigate the mechanism underlying the induction by ECZ of apoptosis and autophagy in mouse colon cancer CT-26 cells. The results showed that ECZ significantly inhibited cell viability in a dose-dependent manner in CT-26 cells. Furthermore, ECZ clearly increased both apoptosis and autophagy via caspase-dependent pathways and AVOs formation in CT-26 cells, respectively. Addition, ECZ induced ROS-mediated apoptosis and autophagy human colon cancer HT-29 cells. Recent findings have suggested that autophagy as a cytoprotective mechanism is closely associated with resistance to apoptosis [8, 30]. In the present study, the inhibition of autophagy by 3-MA enhanced ECZ-induced apoptosis in CT-26 and HT-29 cells. These results showed that autophagy plays a protective role against cytotoxic effects in colon cancer cells via the inhibition of apoptosis.

Many reports have shown that apoptosis and autophagy induction share a common mechanism associated with ROS production [12, 31]. Commonly, ROS play important roles in apoptosis and autophagy signaling and ROS production selectively induces cancer cell death [32]. An autophagy has dual roles to regulate the cell fate in cancer, where autophagy and apoptosis share the cross-talk survival and/or death signal pathway in many cancer cells [32–34]. Other study demonstrated that cross link between autophagy and apoptosis are associated with inhibit cancer cell death by autophagy inhibitors and antioxidants using potentiate or restore the cytotoxicity effect of the drug [35, 36]. Consistent with these previous reports, the present study showed that ECZ-induced apoptosis and autophagy in CT-26 cells were significantly reversed by pretreatment with the ROS scavenger NAC. Treatment with NAC also restored the expression of the apoptosis- and autophagy-related proteins procaspase-3 and LC3. Furthermore, the inhibition of autophagy enhanced ECZ-induced apoptosis in CT-26 cells, and this was further enhanced by ROS production following treatment with 3-MA. These results demonstrated that intracellular ROS production plays a critical role during ECZ-induced apoptosis and autophagy and that increasing ROS generation by the inhibition of autophagy results in enhanced apoptosis.

## Conclusion

The results of this study showed that ECZ induced apoptosis in mouse colon cancer CT-26 cells via caspase-dependent pathways and that it triggered autophagy via an increase in the formation of autophagosomes. In particular, we showed that ROS production played a role in ECZ-induced apoptosis and autophagy. The inhibition of autophagy enhanced ECZ-induced apoptosis resulting from increased ROS production, thus suggesting that autophagy may play a cytoprotective role via apoptosis resistance. Therefore, we believe that ECZ can be used for cancer chemoprevention or as a chemotherapeutic agent; however, further studies are needed to explore the molecular mechanisms and evaluate the anticancer activity of ECZ in vivo.

## Supplementary information

**Additional file 1: Figure S1.** Ethanol extracts of Chrysanthemum zawadskii Herbich (ECZ) induced reactive oxygen species (ROS)-mediated apoptosis and autophagy in human colon cancer HT-29 cells. (A) Apoptosis induction. (B) N-Acetyl-L-cysteine (NAC) attenuated apoptosis induced by ECZ. (C) Detection of acidic vesicular organelles (AVOs). (D) Effects of NAC on autophagy. Cells were treated with various concentrations (100–300 μg/ml) of ECZ for 24 h and stained with annexin V/propidium iodide and 1 μM acridine orange (AO) at 37 °C in the dark for 20 min and then analyzed by flow cytometry. Cells were pretreated with 1 mM NAC prior to 1 h and annexin V/propidium iodide and AO-stained cells were evaluated by flow cytometry. Data are presented as the mean ± standard deviation for at least three independent experiments. ***p < 0.001 vs. the control group; ^###^p < 0.001 vs. the ECZ treated group.

**Additional file 2: Figure S2.** Inhibition of autophagy enhanced apoptosis induced by ethanol extracts of Chrysanthemum zawadskii Herbich (ECZ) in human colon cancer HT-29 cells. (A) Pretreatment with 3-methyladenine (3-MA) reversed the ECZ-induced increase in autophagy. (B) Treatment with 3-MA enhanced ECZ-induced apoptosis. Cells were pretreated with 5 mM 3-MA prior to 1 h. Apoptotic and autophagic cells were evaluated by flow cytometry. Data are presented as the mean ± standard deviation for at least three independent experiments. ***p < 0.001 vs. the control group; ^###^p < 0.001 vs. the ECZ treated group.

## Data Availability

The datasets for supporting the outcomes of the study are included in the article. However, additional information can be provided on request made to the corresponding author.

## Abbreviations

3-MA: 3-methyladenine
AO: acridine orange
ATCC: American Type Culture Collection.
AVOs: acidic vesicular organelles
CCK-8: cell counting kit-8
CZ: *Chrysanthemum zawadskii* Herbich
DCF-DA: dichlorodihydrofluorescein diacetate
DMEM: Dulbecco’s modified Eagle’s medium
ECZ: ethanol extracts of *Chrysanthemum zawadskii* Herbich
FBS: fetal bovine serum
HPLC: High performance liquid chromatography
LC-3B: microtubule-associated protein 1 light chain-3B
NAC: N-acetyl-L-cysteine
PARP: poly (ADP-ribose) polymerase
PI: propidium iodide
ROS: reactive oxygen species
