# Supplementary figures and images for: Ethanol extract of Chrysanthemum zawadskii Herbich induces autophagy and apoptosis in mouse colon cancer cells through the regulation of reactive oxygen species
Source: BMC Complement Altern Med. 2019 Oct 21;19:274. doi: 10.1186/s12906-019-2688-0 (PMC6805551; doi:10.1186/s12906-019-2688-0)

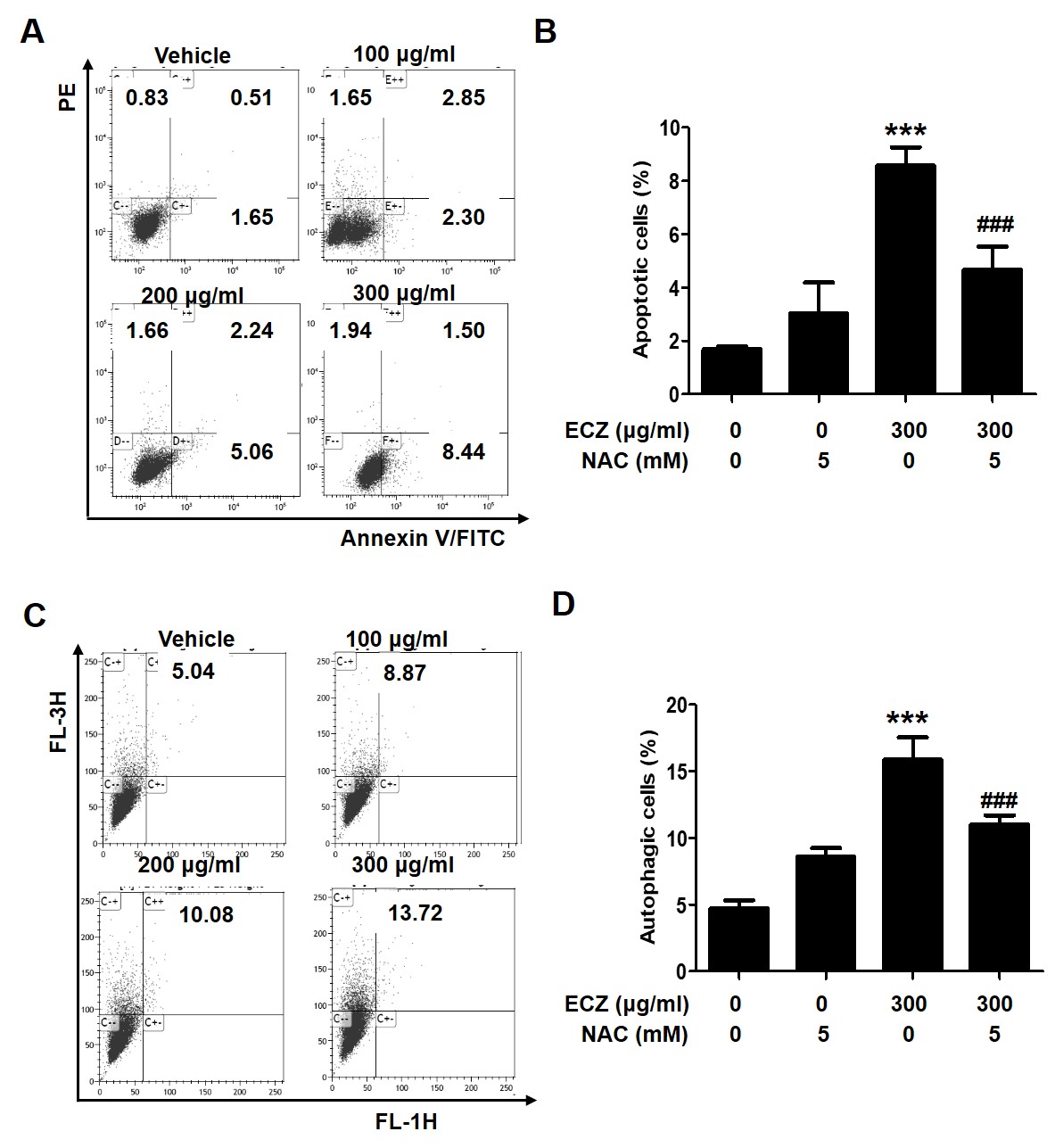

Supplement: Supplementary file 1 — Additional file 1: Figure S1. Ethanol extracts of Chrysanthemum zawadskii Herbich (ECZ) induced reactive oxygen species (ROS)-mediated apoptosis and autophagy in human colon cancer HT-29 cells. (A) Apoptosis induction. (B) N-Acetyl-L-cysteine (NAC) attenuated apoptosis induced by ECZ. (C) Detection of acidic vesicular organelles (AVOs). (D) Effects of NAC on autophagy. Cells were treated with various concentrations (100–300 μg/ml) of ECZ for 24 h and stained with annexin V/propidium iodide and 1 μM acridine orange (AO) at 37 °C in the dark for 20 min and then analyzed by flow cytometry. Cells were pretreated with 1 mM NAC prior to 1 h and annexin V/propidium iodide and AO-stained cells were evaluated by flow cytometry. Data are presented as the mean ± standard deviation for at least three independent experiments. ***p < 0.001 vs. the control group; ###p < 0.001 vs. the ECZ treated group. [file 12906_2019_2688_MOESM1_ESM.jpg]

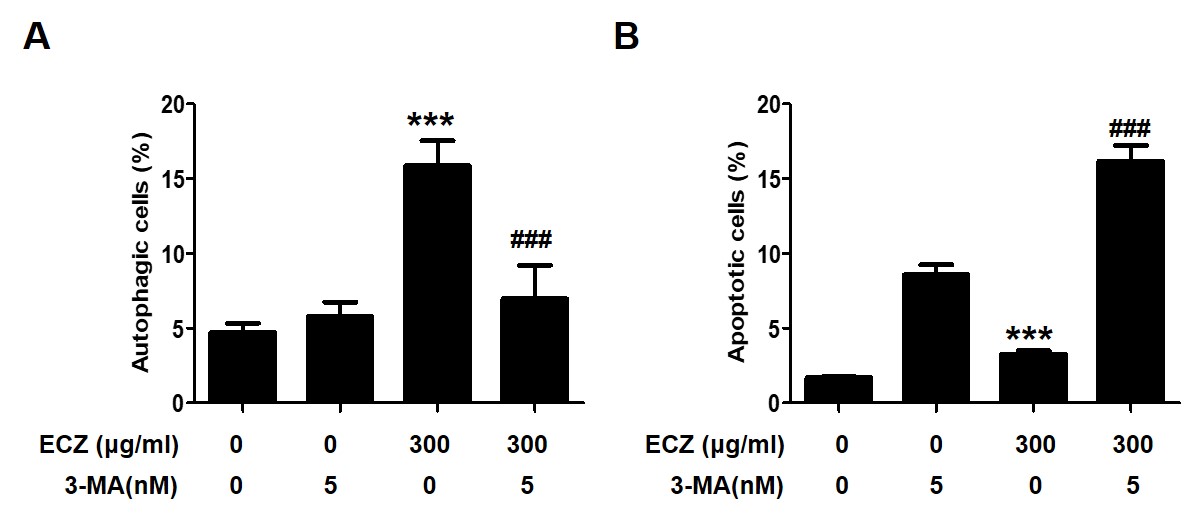

Supplement: Supplementary file 2 — Additional file 2: Figure S2. Inhibition of autophagy enhanced apoptosis induced by ethanol extracts of Chrysanthemum zawadskii Herbich (ECZ) in human colon cancer HT-29 cells. (A) Pretreatment with 3-methyladenine (3-MA) reversed the ECZ-induced increase in autophagy. (B) Treatment with 3-MA enhanced ECZ-induced apoptosis. Cells were pretreated with 5 mM 3-MA prior to 1 h. Apoptotic and autophagic cells were evaluated by flow cytometry. Data are presented as the mean ± standard deviation for at least three independent experiments. ***p < 0.001 vs. the control group; ###p < 0.001 vs. the ECZ treated group. [file 12906_2019_2688_MOESM2_ESM.jpg]
